# Academic independence?

**DOI:** 10.1038/s44319-026-00863-9

**Published:** 2026-07-03

**Authors:** Bernd Pulverer

**Affiliations:** European Molecular Biology Organization, Meyerhofstrasse, Heidelberg, 69117 Germany

**Keywords:** Methods & Resources

## Abstract

US federal policies to establish a ‘gold standard science’ threaten to undermine academic freedom. Individual scientists and institutions must re-articulate why the proposed policies would undermine cultural and economic prosperity and thus ill-serve public interest. At the same time, the scientific community must get its house in order.

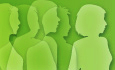

Academic independence is at the heart of scientific research and innovation. The societal contract of academic research regards it as a public good that openly shares objective truths in return for independence from political and economic targets and intervention. The basic concept, along with the need for public funding of research, was famously advocated by Vannevar Bush, head of military research in the USA during WWII, in his 1945 report to the president Science, The Endless Frontier. The policies inspired by his report led to the foundation of the NSF and the US dominance in research and innovation ever since. Globally, the largely self-governing nature of academic research has led to an incredible growth of knowledge in the biosciences—a great cultural accomplishment in itself—and immeasurable medical and economic gains during the past century.

Selective publishing of research findings evolved during the Enlightenment in Europe to provide a robust mechanism for sharing scientific discoveries so that others could build on them; as Isaac Newton quipped: “If I have seen further, it is because I stand on the shoulders of giants.” Later, peer review evolved as a mechanism for objective, expert quality control—a unique achievement of academic research. The system continues to evolve and adapt to the unprecedented rate of scientific discovery seen this century, as Open Science has added preprints and databases to the record of published research. Yet, we still struggle with quality assurance mechanisms and structured platforms to ensure a consistent level of experimental reproducibility fit for Newton’s proclamation. The peer-reviewed publication process has started to risk becoming a victim of its own success as journal selectivity, reduced to misleading metrics, dominates research assessment processes. While research assessment reform is apparent, journal name or quantity still risk trumping quality. Moreover, the biosciences face a reproducibility crisis as a significant proportion of published research in some areas fails straighforward validation.

Unfortunately, these challenges come at a time of unprecedented political and economic pressure on academic research in many parts of the world. Unless resolved, they open an unprotected flank to attacks with the aim of reversing Vannevar Bush’s vision of academic freedom. In addition, data protectionism fueled by a rapidly expanding AI industry and ineffective IP protection across national boundaries risks fragmenting Open Science into geographical domains, exacerbated by concerns about biosecurity and ethics.

Academia’s contract with the public is under scrutiny most explicitly in the USA, where moves for more federal control directly refer back to issues around quality and reproducibility, as expressed in the White House executive order 14303: ‘*Over the last 5 years, confidence that scientists act in the best interests of the public has fallen significantly. A majority of researchers … believe science is facing a reproducibility crisis*’. The directive goes on to define ‘Gold Standard Science’ as: ‘*reproducible; transparent; communicative of error and uncertainty; collaborative and interdisciplinary; skeptical of its findings and assumptions; structured for falsifiability of hypotheses; subject to unbiased peer review; accepting of negative results as positive outcomes; without conflicts of interest*’. So far, so good, but the missive suggests that ‘gold standard science’ will be achieved in the future under direct federal agency control rather than self-regulation by the community. As the head of the US Office of Management Budget (OMB), Russell Vaught, put it ‘[OMB is] *the keeper of commander’s inten*t’. While the US House of Representatives, with bipartisan support, voted for stable research funding in 2026, this has now been framed in a much more restrictive context. Notably, OMB recently released a ‘rule document’ for a brief period of public commenting, which, in its current form, suggests sweeping legislative changes with potentially severe impact on the global biosciences research landscape.

- Peer review is relegated more explicitly to an advisory role to government appointees (‘*provided that peer review recommendations remain advisory and are not … treated as de facto binding by senior appointees*’, section 200.205(d)). While formally this has always been the case, the pointed principles ‘*discretionary awards must, where applicable, demonstrably advance the President’s policy priorities*’ and ‘*must not be used to* …*compromise public safety or promote anti-American values’* and *‘applicants should commit to complying with administration policies, procedures, and guidance respecting Gold Standard Science’*, imply that funding decisions would be made primarily to address government priorities, opening the door to political interference. Laudably, the guidance also notes that ‘*agencies should prioritize an institution’s commitment rigorous, reproducible scholarship’*.
- International cooperation would require case-by-case approval by government agencies (‘*Federal agencies may not issue Federal awards … to foreign entities except where expressly authorized by statute or where … determined by the agency’s senior appointee’* (Section 200.202(e)) and would be largely prohibited for ‘*covered foreign countries*, including China (Section 200.220). Note that federal agencies such as USDA are already curtailing international cooperation and publication: ‘*publications shall not be published in journals … whose publisher has editorial offices managed, owned, controlled, or physically or virtually located in, or operated out of, a foreign country of concern*’ (USDA Departmental regulation DR 1020-006, section 5)
- Publishing—in both open access or subscription journals —would not be supported by federal funding (‘*OMB is revising the section to make publication costs unallowable unless approved in advance by the Federal agency on a case-by-case basis…publication costs (including page charges, article processing charges (APCs)…) are unallowable under Federal awards*’, as ‘*Publication costs are not inherently necessary to carry out the core programmatic objectives…*’ Section 200.461). As universities rely on federal grants for both direct project funding and overhead recovery, academic libraries may no longer be able to recover subscription expenses through grants.
- Conference attendance would have to be applied for with grant applications so far ahead of the event as to render it unrealistic (‘*the costs for attending conferences are allowable only if participation in the conference is expressly approved by the Federal agency and included in … the Federal award*’ Section 200.432). While federal office holders were previously subject to such restrictions, this would thus be extended to fundees.
- Grants can be halted for up to 90 days or suspended by the federal agency ad hoc (‘*terminate active grants at the discretion of the Federal agency … if a Federal award does not effectuate program goals, Federal agency priorities, or the national interest*’ Section 200.340 and 200.341)

As Holden Thorp (2026) summarized: “The sweeping new regulations proposed by OMB would subject every federal research funding decision to political review.” US government agencies are obliged to consider public comments before issuing a final ruling: commenting is open until July 13th at https://www.regulations.gov/document/OMB-2026-0034-0001 (see terms at https://www.regulations.gov/user-notice).

Why is this relevant to the global research community? These provisions would largely contain US federal funding within national boundaries and redirect it to priorities defined by the government. If other countries followed suit, the global research enterprise would fragment along national boundaries and political imperatives, severely hampering scientific progress for the good of all.

The silver lining is that the proposed rules may be a wakeup call for the community to demonstrate that it can effectively self-govern to address its problems—otherwise, stress points in the system can and will be used against it. An urgent focus ought to be on enhancing the reproducibility and reliability of reported research findings and on research assessment reform. Rather than lamenting known stress points such as journal selection, publishing charges and lurid examples of integrity breaches, which merely serve to put oil on the fire of political intervention, suggestions for improvements and cooperation on finding solutions are now required.

The hope is that the current attacks on academic freedom galvanize the global research community to cooperate across boundaries of culture and politics to ensure that the gains started in the age of enlightenment do not resolve back to the dark ages. This should include finally putting our house in order. Before that, recall that the mandatory consultation period on the OMB ‘rule document’ closes until July 13. Make use of this important democratic mechanism.
